# Individual, socio-cultural and environmental predictors of uptake and maintenance of active commuting in children: longitudinal results from the SPEEDY study

**DOI:** 10.1186/1479-5868-10-83

**Published:** 2013-06-26

**Authors:** Jenna Panter, Kirsten Corder, Simon J Griffin, Andrew P Jones, Esther MF van Sluijs

**Affiliations:** 1 Medical Research Council Epidemiology Unit,Institute of Metabolic Science, Box 285, Addenbrooke’s Hospital, Hills Road, Cambridge, CB2 0QQ, UK; 2 UKCRC Centre for Diet and Activity Research (CEDAR), University of Cambridge, Box 296, Forvie Site, Robinson Way, Cambridge CB2 0SR, UK; 3 Norwich Medical School, University of East Anglia, Norwich NR4 7TJ, UK

**Keywords:** School, Walking, Cycling, Children, Longitudinal, Behaviour change

## Abstract

**Background:**

Active commuting is prospectively associated with physical activity in children. Few longitudinal studies have assessed predictors of change in commuting mode.

**Purpose:**

To investigate the individual, socio-cultural and environmental predictors of uptake and maintenance of active commuting in 10 year-old children.

**Methods:**

Children were recruited in 2007 and followed-up 12 months later. Children self-reported usual travel mode to school. 31 child, parent, socio-cultural and physical environment characteristics were assessed via self-reported and objective methods. Associations with uptake and maintenance of active travel were studied using multi-level multiple logistic regression models in 2012.

**Results:**

Of the 912 children (59.1% girls, mean ± SD baseline age 10.2 ± 0.3 yrs) with complete data, 15% changed their travel mode. Those children who lived less than 1 km from school were more likely to take up (OR: 4.73, 95% CI: 1.97, 11.32, p = 0.001) and maintain active commuting (OR: 2.80 95% CI: 0.98, 7.96, p = 0.02). Children whose parents reported it was inconvenient to use the car for school travel were also more likely to take up (OR: 2.04, 95% CI: 1.08, 3.85, p = 0.027) and maintain their active commuting (OR: 5.43 95% CI: 1.95, 15.13, p = 0.001). Lower socio-economic status and higher road safety were also associated with uptake.

**Conclusions:**

Findings from this longitudinal study suggest that reducing the convenience of the car and improving the convenience of active modes as well as improving the safety of routes to school may promote uptake and maintenance of active commuting and the effectiveness of these interventions should be evaluated

## Introduction

Physical activity in children is associated with beneficial effects on physical and mental health [1,2]. Despite these benefits, and the existence of guidelines that highlight the importance of being active every day [3], physical activity levels are believed to be insufficient [4,5]. Walking and cycling represent potential ways of achieving recommended levels of daily physical activity [6]. Predominantly cross-sectional evidence suggests that children who walk or cycle to school are more active overall than those who use motorised transport [7,8]. There is also some evidence to suggest that walking and cycling to school (‘active commuting’) is associated with healthier body composition [9].

Reviews suggest that a range of factors from parental attitudes to features of the physical environment influence children’s modes of travel to school [10-12], consistent with ecological models of behaviour [13] and frameworks commonly integrate the wider environmental context of behaviour into the decision-making process around travel choices for children [10,14]. However, the major limitation of this evidence base is that it is predominantly cross-sectional and few studies use longitudinal designs to assess the predictors of changes in walking and cycling [10-12]. One Australian study concluded that neighbourhood social cohesion and road safety, in particular pedestrian crossings, were associated with increases in the number of walking and cycling trips to school [15]. Although one of the first studies in this area, findings may not be applicable to other settings. Furthermore, the study used a relatively small sample and focussed on the predictors of increases in the number of walking and cycling trips irrespective of baseline levels. Levels of physical activity and active commuting are declining among secondary school children [16,17], and interventions to promote active travel have only reported weak effects [18]. It may therefore be important to understand what factors are associated with maintenance as well as uptake of active commuting in order to inform interventions. We therefore aim to contribute to the limited longitudinal evidence in this area, investigating the factors associated with uptake and maintenance of active commuting over 12 months in a sample of 9–10 year-old children living in Norfolk, UK.

## Methods

### Study design, sampling and data collection

The data presented are from the Sport and Physical activity and Eating behaviour: Environmental determinants in Young people (SPEEDY) study, a population-based longitudinal cohort study of children in Norfolk. The study design and sampling have been described in detail elsewhere [19]. Briefly, in 2007, 2064 children (response rate 57%) were recruited from 92 primary schools sampled for environmental heterogeneity. Trained research assistants visited schools between April and July 2007 to administer child questionnaires, take physical measurements and distribute a home pack, including a parent questionnaire, to be returned to school one week later. Invitations were posted to all 2064 initial participants one year later (April - July 2008). Those returning a signed parental consent form subsequently received a questionnaire to be returned by mail using an addressed pre-paid envelope. Individual participants were surveyed at approximately the same time of year as baseline to avoid any seasonal differences in behaviour. Ethical approval for this study was obtained from the University of East Anglia ethics committee and all participants gave assent and written parental informed consent.

### Measures

#### Travel mode

At both time points children were asked: “How do you normally travel to school?” with four response categories: “by foot” or “by bike” (active travellers) or “by car” or “by bus or train” (passive travellers). Subsequently, children were classified into one of four groups to reflect changes in commuting over time; (i) used active modes at both time points (maintained active travel), (ii) used passive modes at both time points (maintained passive travel), (iii) switched from passive to active modes of travel (took up active travel) and (iv) switched from active to passive modes of travel (took up passive travel).

#### Individual, socio-cultural and environmental variables

Given the large potential number of personal, social and physical environmental predictors which could have been tested (and accompanying problems of multiple testing), we restricted our exposures to those variables which were: 1) hypothesised to be associated with behaviour from an existing theoretical framework [10]; 2) associated with active commuting in cross-sectional analyses [20,21], and 3) variables relating to parental rules and restrictions that have been shown to be associated with children’s active travel [22]. A total of 31 individual, socio-cultural and environmental variables were included in this analysis, assessed using objective and perceived methods and based on previously used measures [20-27] where possible (see Table 1).

**Table 1:** Description of potential individual, socio-cultural and environmental exposure variables

**Variable Name**	**Description and/or coding**	**Reference**
Individual		
Parent education	Collected in 14 categories then coded as: low, medium, high.	-
Child’s BMI	Children’s height and weight were measured and body mass index computed (weight/height ^2^), which were used to classify children as normal weight, overweight or obese based on internationally recognised cut offs.	[23]
Socio-cultural		
Frequency of children’s non school walking or cycling (p)	Frequency of walking or cycling to either a sports centre, parks, shops or friend’s home using response categories of ‘never’, ‘none within walking or cycling distance’ and four frequency categories ranging from ‘less than once a week’ to ‘6 or more days a week’. Coded as: not walking or cycling to any non-school destination (‘never’ or ‘none within walking or cycling distance’) or any frequency (all other responses).	-
Convenience of the car (p)	Coded as strongly agree and agree and neither, disagree, strongly disagree.	[20]
Parents are around to take their child to school (p)	Coded as strongly agree and agree and neither, disagree, strongly disagree.	
Rules (c)	Sum of responses (‘yes’ or ‘no’) to two items on rules for independent mobility (‘I always have to tell my parents where I am going’ and ‘If I am going out I always have to be back by a certain time’). Score range: 0-2.	-
Peer and parental support (c)	Sum of responses (‘yes’ or ‘no’) to two items on friends and parents encouragement to walk or cycle to school Score range: 0–2.	-
		
Environment		
Perceptions of the neighbourhood environment		
Social cohesion and trust in their neighbourhood (p)	Sum of responses (‘strongly agree’ to ‘strongly disagree’) to seven items regarding social cohesion and trust in neighbourhood*. Summary scores were split into tertiles.	[24]
Physical neighbourhood environment (p)	Sum of responses (‘strongly agree’ to ‘strongly disagree’) to 24-item version of the adapted Neighbourhood Environment Walkability Scale (ANEWS). Summary scores were split into tertiles*.	[20]
Physical neighbourhood environment (p)	Sum of responses (‘strongly agree’ to ‘strongly disagree’) to four statements about the characteristics of the route between home and school (the presence of pavements, cycle-paths, concern about dangerous traffic and concern that something would happen to their child along the route to school). * Summary scores were created and scores dichotomised into ‘low’ or ‘high’ based on the median responses.	[20]
Safety to play in neighbourhood (c)	Child-rated safety to walk or play in the neighbourhood during the day, using ‘yes’ or ‘no’ response categories.	-
Objective measures of the neighbourhood environment		
Road density	Total road lengths divided by neighbourhood area. Scores dichotomised into ‘low’ or ‘high’.	[21,25]
Proportion of primary roads	Length of primary (A) roads divided by total road length. Scores dichotomised into ‘low’ or ‘high’.	[21,25]
Streetlights per km of roads	Number of street lights divided by total road length. Scores dichotomised into ‘low’ or ‘high’.	[21,25]
Effective walkable area	Total neighbourhood area (the area that can be reached via the street network within 800 m from the home) by the potential walkable area (the area generated using a circular buffer with a radius of 800 m from the home). Scores dichotomised into ‘low’ or ‘high’.	[21,25]
Connected node ratio	Number of junctions divided by number of junctions and cul-de-sacs. Scores dichotomised into ‘low’ or ‘high’.	[21,25]
Junction density	Number of junctions divided by total neighbourhood area. Scores dichotomised into ‘low’ or ‘high’.	[21,25]
Land-use mix	Proportion of each land use^1^ squared and summed. This score is also known as the Herfindahl-Hirschman Index. Scores dichotomised into ‘low’ or ‘high’.	[21,25]
Deprivation	Index of multiple deprivation scores for neighbourhood of home address. Scores split into quartiles.	[21,25]
Urban–rural status	Urban rural status of home address. Classification of Bibby and Shepherd (2006) and coded into: urban, town and fringe and other.	[21,25]
Objective measures of the route environment		
Distance between home and school	Shortest route between home address and nearest school access point. Coded as more than 2 km, 1-2 km or less than 1 km.	[21,25]
Streetlights per km of route	Streetlights within 100 m of route divided by route length. Scores dichotomised into ‘low’ or ‘high’.	[21,25]
Presence of a main road en route	Presence of primary (A) road as part of route. Coded as ‘yes’ or ‘no’.	[21,25]
Route length ratio	Route length divided by the straight line distance between the home and school. Coded as: indirect route (≥1.6) or direct route (<1.6) This is sometimes known as the route directness index.	[21,25,26]
Land-use mix along the route	Percentage of each land use^1^ within 100 m of route squared and summed. Scores dichotomised into ‘low’ or ‘high’. This score is also known as the Herfindahl–Hirschman index.	[21]
Route within an urban area	Percentage of route which passes through urban area. Coded as: ‘yes’ (route is completely within an urban area) or ‘no’ (route not completely within an urban area).	[21]
Objective measures of the school environment		
School travel plan	Head teachers reported whether their school had a school travel plan (‘yes’ or ‘no’) (a formal document, which identifies ways of encouraging more children to walk, cycle or use public transport to get to school).	[27]
Held walk to school campaigns	Head teachers reported whether they held walk to school campaigns (including walk to school days or weeks) (‘yes’ or ‘no’).	[27]
Walking provision	School audit assessment of the facilities within and surrounding the school for walking. Scores dichotomised into quartiles.	[27]
Cycling provision	School audit assessment of the facilities within and surrounding the school for cycling. Scores dichotomised into quartiles.	[27]

(p) parent-reported measures (c) child-reported measure.

^1^Seventeen different land uses were classified: farmland, woodland, grassland, uncultivated land, other urban, beach, marshland, sea, small settlement, private gardens, parks, residential, commercial, multiple-use buildings, other buildings, unclassified buildings, and roads.

* If summary scores were computed comprising seven or more items and more than two-thirds of the items were completed, the answers for the remaining items were imputed with the most conservative scores. Otherwise summary scores were set to missing.

Neighbourhood environment is defined using an 800 m network based buffer around the home location and route environment is defined using a 100 m network based buffer around the route.

### Data analyses

Descriptive data were summarized using percentages and chi-squared tests. Simple unadjusted associations between individual, socio-cultural, and physical environmental factors and change in travel modes were assessed using multilevel logistic regression, to allow for clustering at the school level. Analyses were stratified by baseline commuting mode to assess i) the odds of taking up active commuting compared to maintaining passive travel and ii) the odds of maintaining active commuting compared to taking up passive travel. A number of variables showed correlations with each other and therefore to avoid problems of multicollinearity, where correlations existed, the variable most strongly associated with behaviour was selected for multivariable analysis. Age and sex (both hypothesised a priori to be associated with active travel) and variables that were associated with the outcome in the simple analyses (p < 0.25) were included in multiple multilevel regression models [28]. All analyses were carried out using Stata 11.1.

## Results

Of the 2064 children who participated at baseline, 1267 (61.3%) responded and 999 returned completed questionnaires. Of these, 912 (44.2% of the original sample) provided information on travel mode at both time points and were included in this analysis. These children were more likely to be girls (59% v 51%, *p* = 0.03), to live in an urban location (35% v 29%, *p* = 0.003) and their households were more likely to have access to a car (96% v 93%) and own their own home (78% v 69%) than those excluded (both *p* = 0.001). There were no differences in baseline weight status and travel mode to school between those included and excluded.

Table 2 shows the characteristics of the sample. 15.3% of children reported a change in usual travel mode to school between baseline and follow-up. Of these 9.5% (n = 87) took up active commuting and 5.8% (n = 53) took up passive commuting, whilst 40.1% (n = 366) reported maintaining passive travel modes on the commute at both time points and 44.6% (n = 406) children maintained their active commuting at both time points. Between baseline and follow-up 42 (4%) children moved home. We repeated the analysis with and without these children excluded and the final results did not differ substantially and therefore we present results including these children. None of the children moved school.

**Table 2:** Baseline characteristics of the SPEEDY sample for those with valid data on travel mode to school at both baseline and follow-up

	**Percentage (n) (n =912)**
Child characteristics	
Mean age (SD)	10.23 (0.30)
Gender	
Male	40.9 (373)
Female	59.1 (539)
Travel mode to school	
Passive - car	43.0 (392)
Passive - train or bus	6.7 (61)
Active - walk	40.8 (372)
Active - cycle	9.5 (87)
Weight status	
Normal	78.63 (714)
Overweight	16.96 (154)
Obese	4.41 (40)
Parental characteristics	
Age left full time education	
<16 years	44.7 (391)
16-18 years	21.5 (188)
Over 18 years	33.7 (295)
Household characteristics	
Housing tenure	
House owner	78.6 (694)
Renting	21.4 (189)
Car access	
No	3.6 (32)
Yes	96.4 (853)
Urban rural status	
Village and hamlet	32.0 (648)
Town and fringe	28.4 (575)
Urban area	39.5 (800)
Area-level deprivation score	
Least deprived	17.3 (156)
2^nd^ quartile	38.7 (348)
3^rd^ quartile	24.2 (218)
Most deprived	19.8 (179)
Mean distance to school in km (SD)	2.6 (3.78)

Initial examination of the objectively measured neighbourhood and route measures showed that some were strongly correlated with each other. Neighbourhood street light density, proportion of roads, land use mix and urban–rural status were all correlated (all *p* < 0.01) and following the pre-specified procedures, urban–rural status was retained. The density of junctions in the neighbourhood and the effective walkable area were also correlated whilst route length and urban–rural status of route were associated with each other (*p* < 0.01), resulting in only route length and effective walkable area being used. In unadjusted analyses of initially passive commuters who switched to active modes (Table 3), a range of child and parent characteristics, measures of the social environment, and of the physical environment (n = 17) were associated with uptake of active commuting. The final adjusted regression models showed that children whose parents had lower levels of education, reported higher levels of safety on the route between home and school, for whom it was inconvenient to take their child to school by car, lived in urban areas, and lived within 1 km of school were more likely to take up active commuting. Those children whose routes to school were more direct were less likely to switch to active commuting.

**Table 3:** Logistic regression analyses exploring the odds of taking up active travel

		**Minimally adjusted model OR**		**Maximally adjusted model OR**	
		**(95% CI)**	**p**	**(95% CI)**	**p**
Child characteristics	Gender				
	Female (reference: male)	1.19 (0.74, 1.92)	0.481	1.22 (0.65, 2.27)	0.532
	Weight status				
	Overweight/Obese (reference: normal)	0.83 (0.46, 1.49)	0.538	n.e	
Parent characteristics	Age parent/carer left full-time education (reference: >16 yrs)				
	< 16 years	**2.10 (1.31, 3.37)**	**0.002**	**2.23 (1.20, 4.13)**	**0.011**
Social environment for active commuting	Children walk or cycle to non-school destination at least once a week (reference: no)				
	Yes	**2.01 (1.21, 3.34)**	**0.007**	1.63 (0.82, 3.22)	0.160
	Composite rules score (reference: no rules)*				
	Mid (score = 1)	0.77 (0.19, 3.12)	0.770	n.e	
	High (score = 2)	0.91 (0.25, 3.36)			
	Convenient to take the car (reference: Strongly agree/ Agree/Neither agree nor disagree)				
	Strongly disagree/ Disagree	**2.07 (1.28, 3.36)**	**0.003**	**2.04 (1.08, 3.85)**	**0.027**
	Around to take child to school (reference: Strongly agree/ Agree/Neither agree nor disagree)				
	Strongly disagree/ Disagree	0.88 (0.46, 1.68)	0.691	n.e	
	Parental and peer encouragement (reference: neither)				
	Parental or peer encouragement for AT	**1.86 (1.08, 3.20)**		1.22 (0.61, 2.44)	
	Parental and peer encouragement for AT	**2.36 (1.26, 4.41)**	**0.001**	1.01 (0.44, 2.31)	0.879
Physical environment for active commuting	*Perceptions of the environment*				
	Composite of route safety (reference: low)				
	High	**4.61 (2.79, 7.64)**	**0.001**	**2.67 (1.42, 5.03)**	**0.002**
	Neighbourhood social cohesion score (reference: low)				
	High	0.81 (0.51, 1.29)	0.372	n.e	
	Neighbourhood environment score (reference: low)				
	High	**1.96 (1.22, 3.15)**	**0.005**	0.96 (0.50, 1.82)	0.891
	Safe to walk/play alone in my neighbourhood during the day (reference: no)				
	Yes	1.14 (0.68, 1.91)	0.605	n.e	
	*Objective environment Neighbourhood*				
	Urban rural status (reference: village and hamlet)				
	Town and fringe	**5.24 (2.72, 10.09)**		**2.89 (1.13, 7.40)**	
	Urban	**5.76 (3.04, 10.91)**	**0.001**	**4.18 (1.41, 12.40)**	**0.013**
	Deprivation (reference: Least deprived)				
	2^nd^ quartile	0.83 (0.39, 1.77)		n.e	
	3^rd^ quartile	0.57 (0.24, 1.33)			
	Most deprived	1.27 (0.56, 2.90)	0.570		
	Road density (reference: low)				
	High	**4.98 (2.99, 8.31)**	**0.001**	n.i	
	Street light density (reference: low)				
	High	**5.54 (2.84, 10.78)**	**0.001**	n.i	
	Junction density (reference: low)				
	High	**0.51 (0.32, 0.82)**	**0.005**	n.i	
	Effective walkable area (reference: low)				
	High	**0.48 (0.30, 0.78)**	**0.003**	2.00 (0.96, 4.15)	0.063
	Land use mix (reference: low mix)				
	High mix	**0.43 (0.26, 0.69)**	**0.001**	n.i	
	*Objective route environment*				
	Route length between home and school (reference: >2km)				
	1-2km	**6.56 (3.61, 11.91)**		**3.32 (1.52, 7.25)**	
	<1km	**13.25 (6.75,25.99)**	**0.001**	**4.73 (1.97, 11.32)**	**0.001**
	Main road en route (reference: yes)				
	No	**0.44 (0.26, 0.72)**	**0.001**	0.73 (0.35, 1.53)	0.403
	Route to school completely within an urban area (reference: no)				
	Yes	**8.66 (5.09, 14.75)**	**0.001**	n.i	
	Streetlight density on route (reference: low <4%)				
	High (<4%)	**2.48 (1.52, 4.03)**	**0.001**	1.33 (0.60, 2.95)	0.482
	Route length ratio (reference: low)				
	High	**0.35 (0.20, 0.58)**	**0.001**	**0.41 (0.19, 0.89)**	**0.024**
	*Objective school environment*				
	Walk to school initiative (reference: no)				
	Yes	0.44 (-0.08, 0.95)	0.103	0.82 (0.40, 1.68)	0.581
	Walking provision (reference: low)				
	High	1.43 (0.86, 2.38)	0.242	0.89 (0.39, 2.02)	0.783
	Cycling provision (reference: low)				
	High	1.56 (0.98, 2.52)	0.063	0.99 (0.50, 1.97)	0.981

Reference category is those children who reporting using passive modes of travel at both times. Number of children included in the final model is 400, due to missing values in some explanatory variables. Bold font indicates variable significant at p < 0.05. Where one p-value is reported for several categories, it refers to a test for trend across the groups. n.e = not entered into model, n.i = not included in final model due to collinearity with other variables.

In unadjusted analyses of initially active commuters (Table 4), fewer potential predictors (n = 4) were associated with maintaining active commuting. In the final adjusted regression models, those children who lived less than 1 km from school and whose parents reported that it was inconvenient to take their child to school by car were more likely to maintain their active commuting behaviour.

**Table 4:** Logistic regression analyses exploring the odds of remaining an active traveller

		**Minimally adjusted model OR**		**Maximally adjusted model OR**		
		**(95% CI)**	**p**	**(95% CI)**		**p**
Child characteristics	Gender					
	Female (reference: male)	0.96 (0.53, 1.72)	0.887	0.88 (0.45, 1.72)		0.716
	Weight status					
	Overweight/Obese (reference: normal)	0.62 (0.33, 1.19)	0.152	0.60 (0.29, 1.24)		0.165
Parent characteristics	Age parent/carer left full-time education					
	< 16 years (reference: >16 years)	1.09 (0.61, 1.94)	0.765	n.e		
Social environment for active travel	Children walk or cycle to non-school destination at least once a week (reference: no)					
	Yes	1.44 (0.78, 2.68)	0.246	1.42 (0.69, 2.91)		0.338
	Composite rules score (reference: no rules)*					
	Mid (score = 1)	0.90 (0.10, 8.01)	0.481	n.e		
	High (score = 2)	0.68 (0.09, 5.40)				
	Convenient to take the car (reference: yes)					
	No	**10.74 (5.03, 22.92)**	**0.001**	**5.43 (1.95, 15.13)**		**0.001**
	Around to take child to school (reference: no)					
	Yes	1.08 (0.56, 2.07)	0.823	n.e		
	Parental and peer encouragement (reference: neither)					
	Parental or peer encouragement for AT	0.87 (0.41, 1.85)				
	Parental and peer encouragement for AT	1.14 (0.50, 2.59)	0.754	n.e		
Physical environment for active commuting	*Perceptions of the environment*					
	Route safety (reference: low)					
	High	**2.05 (1.06, 3.98)**	**0.032**	1.49 (0.66, 3.37)		0.334
	Neighbourhood social cohesion score (reference: low)					
	High	1.41 (0.80, 2.51)	0.238	1.73 (0.90, 3.33)		0.102
	Neighbourhood environment score (reference: low)					
	High	1.32 (0.74, 2.36)	0.345	n.e		
	Safe to walk/play alone in my neighbourhood during the day (reference: no)					
	Yes	1.14 (0.60, 2.17)	0.669	n.e		
	*Objective neighbourhood environment*					
	Urban rural status (reference: village and hamlet)					
	Town and fringe	1.45 (0.69, 3.05)				
	Urban	1.29 (0.63, 2.62)	0.486	n.e		
	Deprivation (reference: Least deprived)					
	2^nd^ quartile	0.50 (0.22, 1.18)				
	3^rd^ quartile	0.61 (0.24, 1.57)				
	Most deprived	0.60 (0.23, 1.57)	0.473	n.e		
	Road density (reference: low)					
	High	1.59 (0.89, 2.82)	0.116	1.15 (0.57, 2.33)		0.693
	Street light density (reference: low)					
	High	1.12 (0.55, 2.27)	0.762	n.e		
	Junction density (reference: low)					
	High	1.00 (0.56, 1.78)	0.996	n.e		
	Effective walkable area (reference: low)					
	High	0.99 (0.56, 1.76)	0.977	n.e		
	Land use mix (reference: low mix)					
	High mix	1.26 (0.70, 2.26)	0.449	n.e		
	*Objective route environment*					
	Route length between home and school (reference: >2km)					
	1-2km	**2.46 (1.12, 5.43)**		**1.24 (0.46, 3.39)**		
	<1km	**6.32 (2.89, 13.85)**	**0.001**	**2.80 (0.98, 7.96)**		**0.020**
	Main road en route (reference: yes)					
	No	0.92 (0.46, 1.83)	0.814	n.e		
	Route to school completely within an urban area (reference: no)					
	Yes	**2.71 (1.48, 4.95)**	**0.001**	1.12 (0.49, 2.55)		0.790
	Streetlight density on route (reference: low <4%)					
	High (<4%)	0.80 (0.45, 1.42)	0.439	n.e		
	Route length ratio (reference: low)					
	High	0.85 (0.46, 1.56)	0.602	n.e		
	*Objective school environment*					
	Walk to school initiative (reference: no)					
	Yes	1.23 (0.69, 2.21)	0.470	n.e		
	Walking provision (reference: low)					
	High	1.41 (0.77, 2.58)	0.258	n.e		
	Cycling provision (reference: low)					
	High	1.03 (0.57, 1.87)	0.911	n.e		

Reference group is those children who switched from active to passive modes of travel. Number of children included in the final model is 419, due to missing values in some explanatory variables. Bold font indicates variable significant at p < 0.05. Where one p-value is reported for several categories, it refers to a test for trend across the groups. n.e = not entered into model, n.i = not included in final model due to collinearity with other variables.

## Discussion

### Principal findings

We found that around 15% of children changed their usual travel mode in their final years at primary school, indicating that travel choices are relatively stable in this age group. Children were more likely to take up or maintain active travel if they lived closer to school and if their parents reported that they thought it inconvenient to use the car for school travel. For uptake of active commuting, further individual, socio-cultural and environmental predictors were associated, including lower socio-economic status and higher road safety. These results indicate that a combination of environment and convenience play an important role in predicting active commuting longitudinally.

### Comparability with other studies

In general, we found few social and physical environmental predictors were associated with children’s change in travel in mode to school and this is consistent with the findings of Hume et al. [15] who report that few social and physical environmental predictors were associated with increases in children’s and adolescent’s active commuting. However, distance to school was positively associated with both maintenance and taking up active commuting, which is consistent with cross-sectional evidence indicating a strong consistent association between distance and commuting mode [10]. Regardless of individual or socio-cultural characteristics, children may be unlikely to take up or maintain levels of walking or cycling if the distance is too large and the time taken deemed too long.

Parents’ perceived convenience for using the car for school travel was also associated with both uptake and maintenance of active commuting in this sample. These results mirror our cross-sectional findings [20] and are supported by review-level evidence and frameworks which postulate that parents play a key role in determining the mode of travel used on the journey to school [10,11]. A qualitative review of parents and children’s attitudes to walking and cycling also suggests that car travel is generally perceived as more convenient than walking or cycling and that convenience of modes has a strong influence on the travel mode chosen [29]. Research to date has often failed to consider the potentially complex role parents' decision making processes play in controlling and changing their children's travel behaviours and how environmental characteristics interact with these processes.

A review of predominantly cross-sectional studies concluded that socio-economic status was an important influence on children’s active commuting [30]; children from low socio-economic backgrounds were more likely to actively commute than those from high socio-economic groups. Like that review, our longitudinal results suggest that children whose parents reported lower levels of education were more likely to take up walking and cycling. We were unable to assess whether car ownership was a determinant of change in travel mode here as 96% of our sample lived in households with access to a car. Our results show that the perceived convenience of using the car for the journey to school may particularly important.

Consistent with another study, we also found that direct routes appear to be barriers to children taking up active commuting [31]. In that work, older children with a more direct route to school were less likely to walk or cycle, although those authors observed no such association in children aged 5–6. It is thought that direct routes may discourage active commuting in children, as route directness is often associated with higher levels of traffic flow and more roads to cross, and children may be likely to avoid such areas. This hypothesis is also consistent with our finding that parental perceptions of safety en route to school was positively associated with children’s uptake of active commuting.

### Implications

Children whose parents reported the route between home and school was safe, perceived the car as inconvenient, and lived within 1 km of school were more likely to take up active commuting. Long distances may represent an absolute barrier to uptake and maintenance of active commuting and this has important implications for decisions made by local authorities, for example regarding the location and catchment areas of schools and the development of new residential areas. Our results show that children who live closer to school are more likely to take up active travel, although 30% of children who lived less than 2 km from school used passive modes at follow-up. Hence promoting walking and cycling by other modifiable factors may be important. In those who live further away from school, it may be that children could incorporate walking and cycling into their journeys by actively commuting small sections of the journey, rather than the entire journey, for example by walking or cycling the last part of the journey to school. These approaches are in line with recommendations to promote physical activity in children produced by National Institute for Health and Clinical Excellence [32]. Our findings and those of others [29] suggest that interventions which focus on road safety may also be particularly efficacious. Few intervention studies account for the distance children have to travel to get to school in their design or analyses and we recommend that future research does so. Equally other factors such as reach, dose and timing of interventions are also crucial. Our finding that socio-cultural and physical environmental factors are associated with uptake of active commuting suggest that provision of more supportive social and physical environments for active commuting may be an important intervention strategy. It is possible that these changes to the physical environment may result in changing social environments for active commuting, such as perceptions about convenience.

Given that few high quality intervention studies to promote active commuting have been conduced and many have produced trivial or small effects [18], further longitudinal studies are required in order to inform the development and evaluation of well-designed interventions. Such studies may wish to use a longer time frame (for example from childhood to adolescence) including the transition into high school, which is a period of particular interest given the decline in physical activity which occurs. Longitudinal studies may also wish to investigate the importance of environmental context in different geographical settings ― for example where the provision of infrastructure for cycling is more heterogeneous ―to contribute evidence about the generalisablity of findings from studies on the environment and active commuting.

### Strengths and limitations

This is one of the first studies to examine the predictors of uptake and maintenance of children’s active commuting in the UK. We used a large sample size of predominantly healthy children from urban and rural areas and included a range of potential individual, socio-cultural and environmental predictors of active commuting, including objective and perceived measures pertaining to the neighbourhood, route and school. We conducted surveys at approximately the same time of year at both baseline and follow-up to avoid any seasonal differences in behaviour. Nevertheless, our study has important limitations. Half of the original baseline population (49.4%) consented to take part and only 44.2% of the original sample were included in the analysis. Boys and children from lower socio-economic backgrounds were more likely to be excluded from the analyses. Although no differences were observed for travel mode used at baseline, this differential drop-out and the smaller proportion of obese children originally recruited [19] limits generalisability of our findings. However, the levels of walking and cycling reported here are broadly comparable with other British population based studies [17]. Our sample also contained children within a narrow age range at recruitment and therefore our findings may not be mirrored in younger (who may be influenced by their parents) or older children (who may be influenced by the behaviour of their peers). We assessed changes in travel behaviour to school over one year and only relatively few children had changed their usual travel mode over this time, which may have limited the precision with which we were able to estimate associations. In maximally adjusted models, the associations between change in active commuting and the safety of the route, social cohesion and parental and peer support may have been affected by this. Future studies with increased heterogeneity in change in active travel may want to address these variables in more detail. We also combined travel by public transport and car assigning these children to the passive travel category, although travel by public transport often involves a short walk at either end of the journey. Our modelled routes between home and school were based on the assumption that children would choose the shortest distance, and although these routes may not exactly match the actual routes used, the estimates of distance travelled have been shown to be similar [33].

## Conclusions

In this longitudinal study of 9–10 year olds, we found some support for many of the putative predictors of travel behaviour change suggested by findings from cross-sectional studies and the predictors of uptake and maintenance of active commuting appear to be similar. Interventions to reduce the perceived convenience of car use and improve the convenience of walking and cycling as well as improving road safety may be effective, however further longitudinal and evaluative research is required.

## Competing interest

All authors declare they have no conflict of interest.

## Authors’ contributions

JP and ES conceived of the study and wrote the manuscript. JP conducted the analysis and drafted the manuscript. KC, SG and AJ advised on the design of the study and contributed to the critical revision of the paper. All authors read and approved the final manuscript.
